# In-house digital workflow for virtual surgical planning and guide fabrication in bimaxillary orthognathic surgery with genioplasty: A technical case report

**DOI:** 10.4317/jced.64061

**Published:** 2026-04-25

**Authors:** Martín Andura-Correas, Guillermo Chacón-Ferrer, Marta María Pampín-Martínez, Álvaro Sada-Malumbres, José Luis del Castillo-Pardo-de Vera, José Luis Cebrián-Carretero

**Affiliations:** 1 Department of Oral and Maxillofacial Surgery, Hospital Universitario La Paz, Madrid, Spain

## Abstract

**Background:**

Virtual surgical planning (VSP) has become the standard approach in orthognathic surgery, allowing more precise and predictable skeletal movements. However, many centers still rely on outsourced digital workflows for surgical planning and guide fabrication, which may increase turnaround times and costs. The development of in-house digital workflows offers an alternative that allows full control of the planning and manufacturing process.

**Material and Methods:**

A patient with dentofacial deformity underwent bimaxillary orthognathic surgery (Le Fort I and bilateral sagittal split osteotomy) with guided genioplasty using a "Maxilla First" protocol. DICOM files from CBCT imaging and STL models from intraoral scans were merged in IPS Case Designer to perform virtual surgical planning. Surgical splints and guides were designed using Meshmixer and manufactured in the hospital's 3D laboratory using stereolithography with biocompatible resins. Post-processing included alcohol cleaning, thermal curing, and sterilization according to hospital protocols.

**Results:**

The entire workflow from image acquisition to sterilized surgical guides was completed within approximately 3-5 days. Virtual planning allowed accurate simulation of skeletal movements and fabrication of patient-specific guides and splints. Intraoperative application demonstrated excellent adaptation of the guides and splints, allowing precise transfer of the virtual plan and reducing the need for intraoperative adjustments.

**Conclusions:**

In-house digital workflows represent a feasible and efficient approach for orthognathic surgery planning and guide fabrication. This model improves communication between clinical and technical teams, reduces production times and costs, and increases autonomy in surgical planning.

## Introduction

Virtual surgical planning (VSP) is currently the standard approach in orthognathic surgery, enabling more precise and predictable execution of skeletal movements (1). Most institutions rely on outsourced services that process patient data, design surgical splints, and manufacture guides using 3D printing technologies. Although effective, this model presents several limitations, including longer turnaround times, higher costs, and reduced flexibility for making adjustments (2 , 3). The development of fully in-house digital workflows offers an alternative that provides complete control over the entire process, from image acquisition to final manufacturing (4). This approach enables direct communication between surgeons and technicians, immediate validation of designs, and reductions in both production time and costs. At Hospital Universitario La Paz, an in-house system integrating virtual surgical planning and additive manufacturing has recently been implemented through the hospital's 3D Laboratory, equipped with stereolithography technology and advanced design software. This multidisciplinary environment facilitates collaboration between surgeons and technical staff, improving both precision and efficiency. Recent studies have shown that in-house workflows can achieve accuracy comparable to commercial systems, with mean errors of less than 1 mm (4 - 6). In addition, these workflows enable the fabrication of customized implants and anatomically adapted splints (5 , 7 - 10). The present report describes the application of this hospital-based workflow in a case of bimaxillary orthognathic surgery with guided genioplasty, highlighting its technical feasibility and clinical advantages.

## Case Report

**Figure 1:**
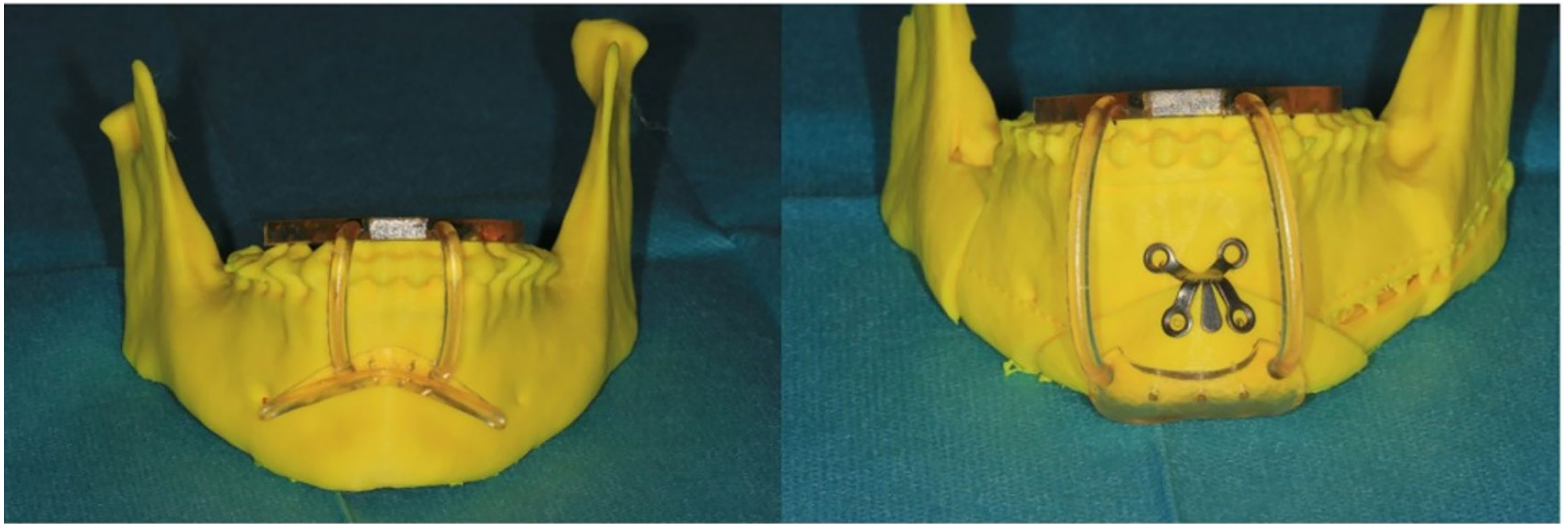
Stereolithographic mandibular models showing the patient-specific cutting guide and positioning guide designed for genioplasty. The fixation plate was pre-bent on the printed anatomical model according to the virtual surgical plan prior to surgery.

A patient with dentofacial deformity presenting facial asymmetry and occlusal cant underwent bimaxillary orthognathic surgery (Le Fort I and bilateral sagittal split osteotomy) with guided genioplasty following a "Maxilla First" protocol. - Data acquisition and virtual planning CBCT imaging was obtained in centric relation and exported as DICOM files. Dental models were obtained through intraoral scanning and exported as STL files. Both datasets were imported into IPS Case Designer software, where anatomical structures were merged, osteotomies were simulated, and movements of the maxilla, mandible, and chin were planned. - 3D editing and design The STL models were refined using Meshmixer for the design of surgical splints, cutting guides, and anatomical models. A cutting guide with tooth-supported anchoring arms and fixation holes was designed to avoid interference with plate placement (Fig. 1a).

1

**Figure 2:**
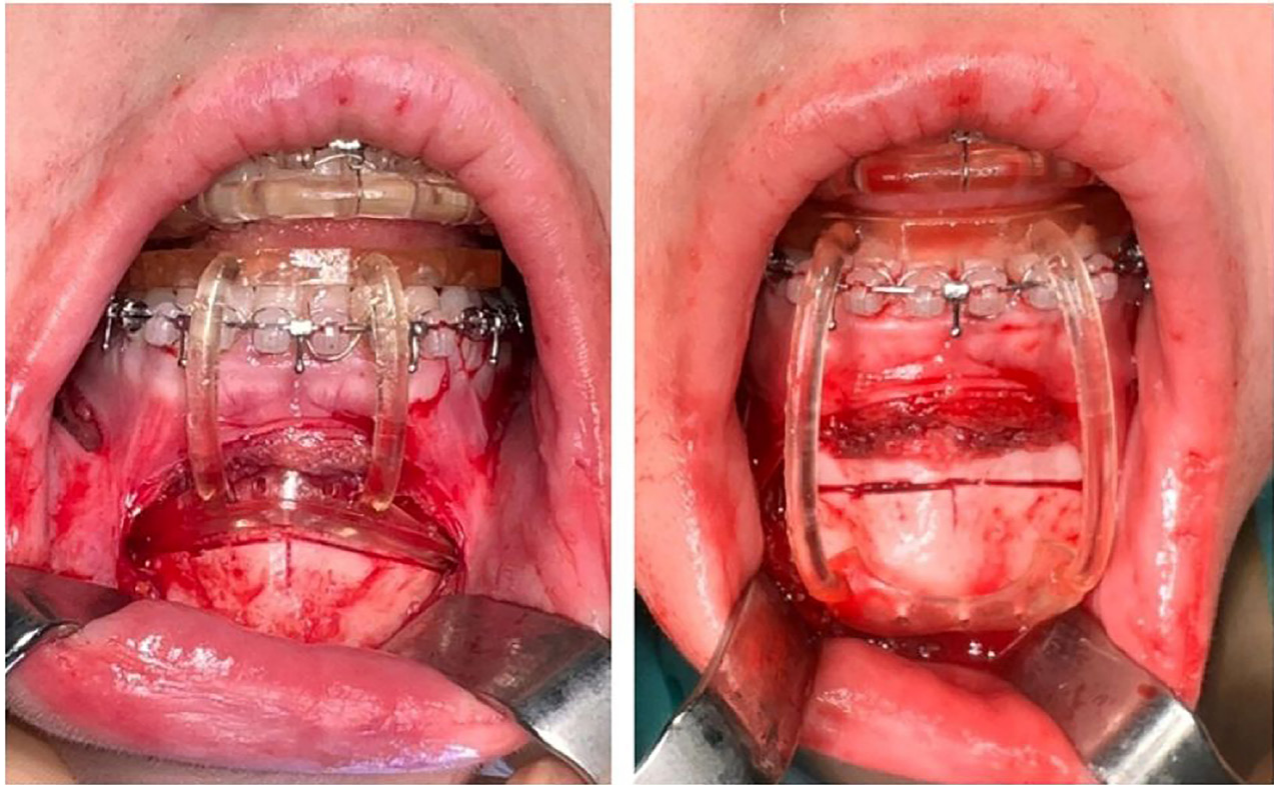
Intraoperative application of the patient-specific cutting and positioning guides during guided genioplasty.

A positioning guide connecting the dental surface with the inferior border of the chin was also designed to ensure accurate repositioning (Fig. 1b). The course of the inferior alveolar nerve was segmented to avoid potential risks during guide design. - Additive manufacturing and post-processing The guides and splints were manufactured in the 3D Laboratory of Hospital Universitario La Paz, accredited under ISO 9001:2015 standards. A stereolithography printer (Form 2, Formlabs, USA) was used. Surgical guides were printed using biocompatible Surgical Guide resin, while splints were printed using Dental LT Clear Resin V2, both with a resolution of 0.05 mm. After printing, the devices were washed in 99% isopropyl alcohol for 20 minutes and post-cured at 60 °C for 30 minutes. Maxillary and mandibular models were printed to verify guide fitting and allow pre-bending of fixation plates according to the virtual plan. All devices were sterilized prior to surgery. Guides were sterilized using ortho-phthalaldehyde (Cidex OPA) for 10 minutes, and anatomical models were sterilized using plasma the day before surgery. - Intraoperative application The surgery was performed following the "Maxilla First" protocol. Pre-bent plates were placed according to the virtual surgical plan, and guided genioplasty was performed using the designed guides and splints, achieving accurate intraoperative adaptation (Fig. 2).

2

## Discussion

The in-house digital workflow described in this report demonstrates an efficient and reproducible approach for orthognathic surgery planning and guide fabrication. One of its main advantages is the ability to maintain full control over the digital workflow, reducing dependence on external providers and significantly shortening production times (4). Previous studies have confirmed the accuracy of virtual surgical planning, reporting deviations of less than 2 mm between planned and postoperative outcomes (6 , 8). Oth et al. (7) demonstrated that in-house printed guides allow precise surgical transfer, while Arcas et al. (5) showed that customized guides may reduce operative time and surgical complications. Another advantage of hospital-based workflows is improved communication between the clinical and technical teams. Direct collaboration between surgeons and technicians facilitates faster decision-making and avoids potential interpretation errors that may occur when planning is outsourced (4 , 5). Although advanced technologies such as augmented reality and CAD/CAM systems have further improved surgical precision (3 , 10), they often require substantial infrastructure and specialized training. The in-house workflow described in this report represents a more accessible and sustainable alternative while maintaining comparable standards of accuracy. However, several limitations must be considered. This approach requires trained technical personnel and certified printing equipment. Furthermore, strict quality control during post-processing and sterilization stages is essential to ensure final accuracy (8). Future studies including three-dimensional postoperative comparisons would help quantify deviations between virtual planning and clinical outcomes. The development of new biocompatible materials and artificial intelligence algorithms may further improve planning accuracy and reduce design times (6). Combined with the increasing availability of hospital-based 3D laboratories, these advances may contribute to the broader adoption of in-house digital workflows in orthognathic surgery.

## Conclusions

The in-house digital workflow enables the integration of all stages of surgical planning and guide fabrication within the hospital environment. This approach provides autonomy, efficiency, and precision while reducing dependence on external services. Although the results obtained in this case demonstrate the feasibility of the system, further prospective studies are required to validate its reproducibility and to compare its clinical and economic performance with outsourced workflows.

## References

[1] Jeon JH (2019). Digital technology in orthognathic surgery: virtual surgical planning and digital transfer. J Korean Assoc Oral Maxillofac Surg.

[2] Pektaş Z, Sönmez TT (2022). Virtual surgical planning in orthognathic surgery with the wafer-only technique versus customized guide-plate system. J Craniofac Surg.

[3] Suenaga H, Sakakibara A, Kawakami H, Taniguchi A, Hoshi K (2025). Computer-Assisted Preoperative Simulation and Augmented Reality for Plate Fixation Positioning in Genioplasty. J Maxillofac Oral Surg.

[4] Locmele P, Radzins O, Lauskis M, Salms G, Slaidina A, Abeltins A (2025). Precision of the Fully Digital 3D Treatment Plan in Orthognathic Surgery. J Clin Med.

[5] Arcas A, Vendrell G, Cuesta F, Bermejo L (2019). Mentoplasty with Customized Guides and Plates Using 3D Technology: a More Precise and Safer Technique. Plast Reconstr Surg Glob Open.

[6] Lee SJ, Yoo JY, Woo SY, Yang HJ, Kim JE, Huh KH, Lee SS, Heo MS, Hwang SJ, Yi WJ (2021). A Complete Digital Workflow for Planning, Simulation, and Evaluation in Orthognathic Surgery. J Clin Med.

[7] Oth O, Mestrallet P, Glineur R (2020). Clinical Study on the Minimally Invasive-Guided Genioplasty Using Piezosurgery and 3D Printed Surgical Guide. Ann Maxillofac Surg.

[8] Alkhayer A, Piffkó J, Lippold C, Segatto E (2020). Accuracy of virtual planning in orthognathic surgery: a systematic review. Head Face Med.

[9] Awad D, Reinert S, Kluba S (2022). Accuracy of Three-Dimensional Soft-Tissue Prediction Considering the Facial Aesthetic Units Using a Virtual Planning System in Orthognathic Surgery. J Pers Med.

[10] Tachizawa K, Sugahara K, Koyachi M, Odaka K, Matsunaga S, Sugimoto M, Katakura A (2025). Enhancing the accuracy of genioplasty using mixed reality and computer-aided design/manufacturing: a randomized controlled trial. Quant Imaging Med Surg.

